# Relationships between Sex and Adaptation to Physical Exercise in Young Athletes: A Pilot Study

**DOI:** 10.3390/healthcare10020358

**Published:** 2022-02-11

**Authors:** Gabriella Pinto, Rosamaria Militello, Angela Amoresano, Pietro Amedeo Modesti, Alessandra Modesti, Simone Luti

**Affiliations:** 1 Department of Chemical Sciences, University of Naples Federico II, 80126 Naples, Italy; gabriella.pinto@unina.it (G.P.); angela.amoresano@unina.it (A.A.); 2 INBB, Istituto Nazionale Biostrutture e Biosistemi, Consorzio Interuniversitario, 00136 Rome, Italy; 3 Department of Biomedical, Experimental and Clinical Sciences “Mario Serio”, University of Florence, 50134 Florence, Italy; rosamaria.militello@unifi.it (R.M.); alessandra.modesti@unifi.it (A.M.); 4 Department of Experimental and Clinical Medicine, University of Florence, 50134 Florence, Italy; pa.modesti@unifi.it; 5 Institute for Sustainable Plant Protection, National Research Council of Italy, 50019 Sesto Fiorentino, Italy

**Keywords:** sport metabolomics, oxidative stress, hormone signalling, adiponectin, basketball

## Abstract

The purpose of this study was to compare the redox, hormonal, metabolic, and lipid profiles of female and male basketball players during the seasonal training period, compared to their relative sedentary controls. 20 basketball players (10 female and 10 male) and 20 sedentary controls (10 female and 10 male) were enrolled in the study. Oxidative stress, adiponectin level, and metabolic profile were determined. Male and female athletes showed an increased antioxidant capacity (27% for males; 21% for females) and lactate level (389% for males; 460% for females) and reduced salivary cortisol (25% for males; 51% for females) compared to the sedentary controls. Moreover, a peculiar metabolite (in particular, amino acids and urea), hormonal, and lipidic profile were highlighted in the two groups of athletes. Female and male adaptations to training have several common traits, such as antioxidant potential enhancement, lactate increase, and activation of detoxifying processes, such as the urea cycle and arachidonic pathways as a response to inflammation. Moreover, we found different lipid and amino acid utilization related to sex. Deeper investigation could help coaches in developing training programs based on the athletes’ sex in order to reduce the drop-out rate of sporting activity by girls and fight the gender stereotypes in sport that also have repercussions in social fields.

## 1. Introduction

During exercise and regular training, several metabolic changes occur in an organism, leading to the activation of adaptive mechanisms aimed at establishing a new dynamic equilibrium, especially at the metabolic level, which guarantees health and better performance in elite athletes [1]. Excessive training and effort could lead to chronic fatigue and muscle damage, with lower performance. Therefore, these adaptive mechanisms are the result of a fine balance between: (i) oxidative stress/inflammation induced by exercise that increases performance and health, and (ii) oxidative stress due to excessive effort that causes fatigue and muscle damage [2]. In this context, the role of oxidative stress and hormone signalling linked to inflammation appears to be crucial for adaptation [2]. Elite athletes have a very strong antioxidant defence system that guarantees free radical detoxification during acute exercise and recovery and, at the same time, the regulation of several hormones ensures adaptation [3,4].

Adiponectin is a signalling hormone with an anti-inflammatory effect, and its plasma level is modified by training [5]. It is a myokine induced by pro-inflammatory mediators (e.g., interferon γ and TNF) [6] produced during training, and it exerts a protective effect against oxidative stress [7]. It seems to play a role in adaptation to physical exercise, acting in an autocrine/paracrine manner. Several studies have indicated that adiponectin plasma concentration is determined by fat mass, with the additional independent effects of age and sex: adiponectin concentration is higher in females and it increases with age [8,9,10]. Some authors have suggested that adiponectin plasma levels are inversely associated with muscle strength [11], and most of this information has been gained through studies on male athletes, while less is known about female athletes. It could be of particular interest to evaluate training responses in relation to sex, since, between male and female athletes, different hormonal, physiological, and muscular statuses exist, which may influence these adaptive mechanisms. Comparison of these mechanisms could broaden our knowledge. Most of the sex-related differences in sport performance are ascribed to androgens; in particular, testosterone and its related metabolite, dihydrotestosterone [12,13,14,15,16]. This sex-based difference is the premise to explain why males acquire larger muscle mass and greater strength, larger and stronger bones, and higher circulating haemoglobin [16]. The observed differences in hormonal, physiological, and muscular status between women and men in physical exercise and adaptation may also be displayed at the metabolic level. There may be sex-independent or sex-dependent traits in adaptations to sport.

Basketball is an intermittent team sport played on a court [17] and characterized by frequent movement changes due to the transition between offense and defence [18]. These transitions differ in terms of movement pattern (e.g., running, jumping, or both), intensity, distance, frequency, and duration, while jumping (unlike in other team sports) approximately every minute [19,20]. Because of these frequent changes during the match, there are periods of high intensity activity interspersed with periods of low or moderate intensity, characterized by aerobic and anaerobic intermittent demands [19].

The aim of the present study is to evaluate plasma oxidative status, adiponectin, cortisol, and steroid hormones related to plasma metabolites and fatty acid levels between female and male basketball players, compared to relatively sedentary controls, with an effort to elucidate the different and common traits between male and female adaptations to physical exercise.

## 2. Materials and Methods

### 2.1. Design

This research was a preliminary cross-sectional study involving a total of 40 subjects: 10 female athletes, 10 female sedentary controls, 10 male athletes, and 10 male sedentary controls. We wanted to highlight the metabolomic, hormonal, and lipidomic profiles of young athletes attributable to training; therefore, we used female and male sedentary people as controls for the physiological differences found within the population. After initial screening, all participants received a complete explanation of the purpose, risks, and procedures of the study. A written informed consent was provided prior to enrolment in the study, which was conducted according to the policy statement set forth in the Declaration of Helsinki, and all the experiments were conducted according to established ethical guidelines. The study was approved by the local ethics committee of the University of Florence, Italy (AM Gsport 15840/CAM_BIO).

### 2.2. Participants

A cohort of 10 female professional basketball players, 10 male professional basketball players, and the respective controls, both 10 females and 10 males, were involved in this study.

The male and female athletes were recruited from the local sports club in Florence (Italy) “US AFFRICO-Firenze” and had been practicing this sport for more than 5 years.

The control groups were recruited among students of the degree course in Motor Sciences, Sport and Health at the University of Florence, and they did not practice any sports. All subjects volunteered to participate following an explanation of all the experimental procedures. They were all adults and of Caucasian ethnicity.

The participants completed a medical history and physical activity questionnaire in order to determine their eligibility. None of them used antioxidant or nutritional supplements. Subjects were selected because of non-smoking status, age, and stable body weight. All females were enrolled randomly with respect to menstrual cycle.

### 2.3. Training Protocol

All players attended the training sessions during the whole season from September until May, and the samples were collected during the twelfth week. The athletes trained at least 5 times a week, according to specific training programs, with sessions lasting 2 h per day. The training protocol involved both technical and aerobic exercise.

Team coaches tracked day-to-day training data for each player and for each training session over the entire season. To assess their adherence to the training plans, the subjects completed the physical activity questionnaires. In particular, we used the International Physical Activity Questionnaires (IPAQ) [21], and the subjects included in category 3, practicing 7 or more days of any combination of walking, moderate-intensity, or vigorous-intensity activities, achieving a minimum of at least 3000 MET minutes/week, were selected. In the present study, the athletic trainers used the rating of perceived exertion (RPE) system of Carl Foster to monitor training load (TL) to obtain the individual responses of athletes in different training sessions [22]. The RPEs of each player were registered across a whole season, and RPE was measured following the completion of each set of exercise and 30 min post-exercise (session RPE).

For both sexes, team coaches followed this classical training program: 30 min of low-moderate running, followed by interval training runs to improve speed and simulate different game situations. Then, as previously reported [23], 3 sets of 12 repetitions of exercises for the shoulder muscles and 3 sets of 15 repetitions of exercises for the abdominals (oblique and the rectus abdominis muscles) were performed.

### 2.4. Materials

Unless specified, all reagents were obtained from Bio-Rad Laboratories (Hercules, CA, USA). Solvents used for the sample preparation and LC–MS/MS analysis have >99.9% purity, as reported by the manufacturing companies. Acetonitrile (ACN) CHROMASOLV was obtained from Honeywell (Charlotte, NC, USA). Methanol (MeOH) and formic acid (HCOOH) were purchased from Sigma (St. Louis, MO, USA).

### 2.5. Procedures

#### 2.5.1. Anthropometric Assessment

All subjects recruited were in the 18–30 age group. Anthropometric measurements were performed at the time of biological samples. Weight was measured to the nearest 0.1 kg and height (H) to the nearest 0.5 cm. All measurements were performed in resting conditions and by the same operator. Body mass index (BMI) was calculated from the ratio of body weight (kg) to body height (m^2^).

#### 2.5.2. Biological Samples

At the twelfth week of training of the athlete groups, a fasting capillary blood sample was taken, using a heparinized microvette (Sarstedt ref. 85.1018), from the forefinger of each volunteer after obtaining written and informed consent. The biological samples (capillary blood and saliva) were collected in morning, 48 h after the last competition and 24 h after the training session. Capillary collection was preferred to venous collection because of its reduced invasiveness, simple execution, and lower cost; moreover, its small volumes of samples were sufficient for the experiments carried out [23].

### 2.6. Plasma Oxidative Stress Measurements

The antioxidant capacity and levels of reactive oxygen metabolites on plasma were determined by using the BAP test (biological antioxidant potential) and the d-ROMs test (derivates of Reactive Oxygen Metabolite). The biomarkers d-ROM and BAP were selected based on their long-term stability. The measurements were carried out according to the manufacturer’s instructions.

Analyses were performed using a free radical analyzer system (FREE Carpe Diem, DIACRON INTERNATIONAL s.r.l, Grosseto, Italy) that included a spectrophotometric device reader and a thermostatically regulated mini-centrifuge. The d-ROM results are expressed in arbitrary units (UCarr), one unit of which corresponds to 0.8 mg/L of hydrogen peroxide. The BAP results are expressed in µmol/L of the reduced ferric ions [24].

### 2.7. Adiponectin Western Blot Analysis

Plasma samples were clarified by centrifugation and total protein contents were obtained using a Bradford assay. An equal amount of each sample (12.5 μg of total proteins) was added to 4 × Laemmli buffer (0.5 M TrisHCl pH 6.8, 10% SDS, 20% glycerol, β-mercaptoethanol, and 0.1% bromophenol blue) and boiled for 5 min. Samples were separated on 12% SDS/PAGE and transferred onto a PVDF membrane using the Trans-Blot Turbo Transfer System (Bio-Rad Laboratories, Hercules, CA, USA). PVDF was probed with primary antibody (Acrp30 Santa Cruz) diluted 1:1000 in 2% milk, and then incubated overnight at 4 °C. After incubation with horseradish peroxidase (HRP)-conjugated antimouse IgG (1:10,000) (Santa Cruz Biotechnology, Dallas, TX, USA)), immune complexes were detected with the enhanced chemiluminescence (ECL) detection system (GE Healthcare, Chicago, IL, USA) and by Amersham Imager 600 (GE Healthcare). For quantification, the blot was subjected to densitometric analysis using the ImageJ 1.53 program. The intensity of the immunostained bands was normalized with the total protein intensities measured by Coomassie brilliant blue R-250 from the same PVDF membrane blot as previously reported [24].

### 2.8. Salivary Cortisol Measurement

The saliva samples of each participant were collected using a salivette cortisol (Sarstedt, Nümbrecht, Germany). All saliva samples were taken at the same hour of the day to avoid any variations due to circadian rhythm. Participants were instructed to not eat, drink, or brush their teeth for 30 min before saliva collection. The cotton sliver of the salivette was taken out and put in the sublingual for 1/2 min, and then put back into the salivette. The samples were centrifuged at 2000 rpm for 2 min. Saliva flow was collected from the salivette’s bottom and stored at −20 °C for further laboratory analysis.

Salivary cortisol was detected using a specific commercial enzyme-linked immunosorbent assay kit (item no. 500360 Cayman chem, Ann Arbor, MI, USA). Saliva samples (50 µL) were used, and the assay was performed according to manufacturer-recommended procedures. The sensitivity provided by the manufacturer is approximately 35 pg/mL, with a detection range from 6.6–4000 pg/mL. The samples were analyzed in duplicate [25].

### 2.9. Gas Chromatography–MS (GC–MS) Analysis of Plasma Metabolites

Metabolite analysis of the plasma samples of the basketball players and control males and females were performed as reported in Militello et al. [23]. Briefly, 100 µL of pooled plasma samples were subjected to methanol/chloroform precipitation and the upper phase was evaporated at room temperature in a rotovapor. The obtained metabolites were analyzed in triplicate by the GC–MS technique after their derivatization with N-trimethylsilyl-N-methyl trifluoroacetamide (MSTFA). The MassHunter data processing tool (Agilent, Santa Clara, CA, USA) was used to obtain a global metabolic profiling using the Fiehn Metabolomics RTL library (Agilent G1676AA).

### 2.10. Extraction of Steroid Hormones and Standard Preparation

An aliquot (50 µL) of plasma from the male and female basketball players and from the control subjects weas added to four volumes of cold MeOH to precipitate the proteins and to extract steroid hormones. After a sonication step of 10 min, samples were centrifuged 10 min at 12,000 rpm at 4 °C, and supernatants were recovered and dried under vacuum.

A similar procedure was used to extract steroid hormones spiked in depleted plasma, in agreement with the guidelines of the certified Eureka kit (https://www.eurekakit.com, accessed on 1 March 2021). A list of nineteen analytes at six levels of concentration enclosed within physiological ranges was included in the datasheet of certified Eureka kit. Standard mixtures were used to perform the quantitative analysis by external standard method.

All dried extracts were then suspended in 50 µL of MeOH containing 0.1% HCOOH for the subsequent LC–MS/MS analysis.

The LC–MS/MS analysis was performed on an AB Sciex QTrap 4000 mass spectrometer, coupled to an ExpressHT™ Ultra HPLC system (Eksigent). Each extract (5 µL), cooled at 4 °C on a refrigerated auto sampler, was injected and separated on a Halo C18 1.0 × 50 mm, 2.7 µm column (at 40 °C) by using a flow rate of 40 μL/min. An aqueous solution containing 5 mM ammonium formate (Sigma Aldrich, Saint Luis, MO, USA) and 0.1% HCOOH was used as a mobile phase, while methanol acidified with 0.1% HCOOH was used as a phase B. A 5 min gradient (0–1 min 30% B, 1–5 min 30–95% B) was applied, with a further 2 min at 95% B to wash the column.

LC–MS/MS analysis was performed in MRM ion mode in positive ion mode (ESI^+^). The instrumental settings included precursor ions (*m*/*z*) and product ions (*m*/*z*), and optimal collision energies as previously reported [24].

### 2.11. Transesterification of Fatty Acid in Plasma Samples

Methanol extracts deprived of the protein component (see above in paragraph “extraction of steroid hormones”) were added with a methanolic solution containing 8% HCl, up to a final HCl concentration of 2%. The reaction of transesterification was performed overnight at 95 °C within a Reacti-Therm™ (Thermo Fisher Scientific™, Waltham, MA, USA) system. At the end of the reaction, the mixture was added to an aqueous solution (1 mL) and fatty acid methyl esters (FAME) were extracted by hexane (1 mL).

The same reaction was performed on a standard mixture containing palmitoleic acid, palmitic acid, linoleic acid, and oleic acid (Sigma Aldrich) for the quantification by external standard method.

After centrifugation, the hexane layer of all samples containing FAMEs was placed into a gas chromatography vial, capped, and directly subjected to a GC analysis performed as reported in Table 1.

### 2.12. Gas Chromatography–Mass Spectrometry (GC–MS) Analysis of Plasma Fatty Acid Methyl Esters (FAMEs)

The gas chromatography (GC) analyses were performed using Agilent GC 6890 coupled with a 5973 MS detector. Each hexane extract (1 µL) was injected into the GC–MS and the analytes were separated on an HP-5 capillary column (30 m × 0.25 mm, 0.25 mM, 5% polisilarilene, and 95% polydimethylsiloxane). Helium was used as the carrier gas at a rate of 1.0 mL min^−1^. The GC injector was maintained at 230 °C, while the oven temperature was held at 60 °C for 3 min, and then increased to 150 °C at 10 °C/min, increasing to 180 °C at 5 °C/min, and finally to 280 °C at 10 °C/min and held for 5 min, for a total separation time of 30 min. The analyzer temperature was kept at 250 °C. The collision energy was set to a value of 70 eV and the fragment ions generated were analyzed at a mass range of 20–500 *m*/*z*.

The identification of each compound was based on the comparison of retention time with the relative standard and fragmentation spectra matching those collected into the NIST 05 Mass Spectral Library.

### 2.13. Statistical Analysis

Data are presented as means +/− standard deviation (SD) from at least three experiments. Statistical analysis was performed by two-way ANOVA (Tukey’s multiple comparisons test) using GraphPad Prism 6.01. The Tukey test compared every mean with every other mean, computing a confidence interval for multiple comparisons of 95% confidence. Significance was defined as *p* < 0.05. We checked the normality and homogeneity distribution of our data by the D’Agostino–Pearson and Brown–Forsythe tests, respectively. For Western blot quantification, the blot was subjected to densitometric analysis using the ImageJ program. As measure of the effect size, we reported the eta-squared (η^2^) calculated in ANOVA (Tukey’s multiple comparisons test) performed on all groups together using GraphPad Prism 8, considering the following intervals: 0.01–0.20 = small effect; 0.21–0.60 = moderate effect; and 0.61–0.99 = high effect.

## 3. Results

### 3.1. Participants’ Characteristics

Descriptive characteristics of the participants as mean ± SD are presented in Table 2. The mean age of the participants was 24.4 ± 4.2 years.

Significant differences emerged between the heights of female basketball players in comparison with the respective controls, and between males and females in both groups (athletes and controls). As expected, males were taller and heavier than females. Despite this, BMI was calculated, and no significant differences were found between the player groups and the control groups (*p* = 0.64 for males, *p* = 1 for females; η^2^ = 0.084) and between males and females (*p* = 0.18 for athletes, *p* = 0.72 for controls; η^2^ = 0.084). Although a significant difference is evident in the age of male athletes compared to the controls, we did not consider it relevant because they were still young males.

Plasma oxidative stress, adiponectin level, metabolite analysis, and salivary cortisol measurements in females were derived from our previous study [23].

### 3.2. Plasma Oxidative Stress and Salivary Cortisol Measurements

**Figure 1:**
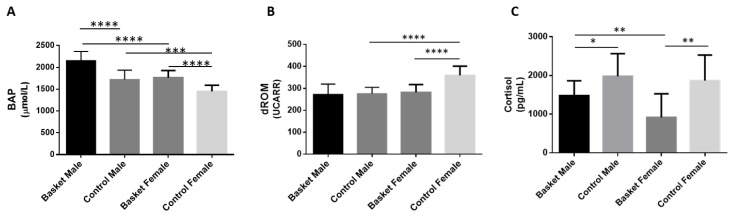
Plasma oxidative stress and salivary cortisol determination. (**A**) The antioxidant capacity was evaluated using the BAP test (biological antioxidant potential). (**B**) The levels of reactive oxygen metabolites using the d-ROM test (derivates of Reactive Oxygen Metabolite) by a free radical analyser system (FREE Carpe Diem, DIACRON INTERNATIONAL s.r.l). (**C**) The cortisol levels measured using an enzyme-linked immunosorbent assay kit. All the measurements (*n* = 10) were performed in triplicate and are reported in the histograms as mean ± SD. Female data are from Militello et al., 2021. The statistical analysis was carried out by two-way ANOVA using GraphPad Prism 8 (* *p* < 0.05; ** *p* < 0.01; *** *p* < 0.001; and **** *p* < 0.0001).

We measured antioxidant capacity (BAP) and the levels of oxidative species (d-ROM) from all the participants, and the results are reported in Figure 1. In a detailed analysis of antioxidant capacity (Figure 1A), we found a significant increase (25.2%, *p* < 0.001; η^2^ = 0.655) in the male basketball players compared to the male control group, and the BAP mean value was 2153.14 ± 211 µmol/L for the athletes in comparison with the controls (1720.33 ± 216 µmol/L). Moreover, a significant increase (21.7%, *p* < 0.001; η^2^ = 0.655) was observed in the female basketball players (1764.7 ± 163 µmol/L) compared to the female control group (1450 ± 139 µmol/L).

Significant differences were also found between males and females in both the basketball and control groups; in particular, male athletes showed an increase (22%; *p* < 0.001; η^2^ = 0.655) compared to female athletes and an analog difference was found between control groups where, in fact, males showed an increase (18.6%; *p* < 0.05; η^2^ = 0.655) compared to females.

Regarding d-ROM values (Figure 1B), the results showed that there is no significant difference between the male athletes (271.9 ± 47 UCarr) and the respective controls (275.5 ± 29 UCarr). On the contrary, the female athletes (281.1 ± 36 UCarr) showed a decrease of d-ROM values of about 21.7% (*p* < 0.0001; η^2^ = 0.452) compared to the female controls (358.8 ± 42 UCarr).

A significant difference was also shown in the control groups between males and females, with a decrease in male subjects of 23.2% (*p* < 0.0001; η^2^ = 0.452) compared to females. The same difference was not found between the male and female basketball groups.

We determined the concentration of salivary cortisol (Figure 1C) and we found a significant decrease (51%; *p* = 0.009; η^2^ = 0.385) in the female basketball group in comparison to the female control group: the cortisol mean value was 1867.68 ± 659.83 pg/mL for female controls and 915.09 ± 613.69 pg/mL for female basketball players. A significant difference was found also in the male groups; in fact, the athletes showed a reduction of 25% (1478.3 ± 385 pg/mL, *p* = 0.017; η^2^ = 0.385) compared to the control values (1979.65 ± 583.9 pg/mL).

There was no significant difference between control groups, but between the athlete groups, the male basketball players had shown an increase of 61.5% (*p* = 0.004; η^2^ = 0.385) in comparison with the female athletes.

### 3.3. Plasma Adiponectin Determination

**Figure 2:**
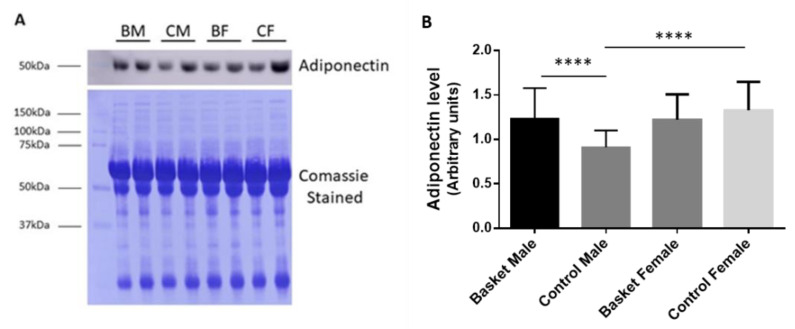
Plasma adiponectin levels. (**A**) A representative immunoblot of adiponectin with the corresponding Coomassie-stained PVDF membrane. (**B**) Relative quantification of adiponectin carried out by the Image J program. Female data are from Militello et al., 2021. Statistical analysis was performed by two-way ANOVA (Tukey’s multiple comparisons test) using GraphPad Prism 8. Bars represent the mean ± SD (*n* = 10; **** *p* < 0.0001).

The plasma adiponectin level was determined by Western blot analysis as previously reported [23]. In the female groups, no significant differences were found between athletes and controls. Regarding the male groups, adiponectin levels showed a significant increase of 35.4% (*p* < 0.0001, η^2^ = 0.230) in basketball players in comparison with the respective controls (Figure 2). In addition, between the controls, we observed a significant decrease of 31.4% (*p* < 0.0001, η^2^ = 0.230) in males compared to females.

### 3.4. GC–MS Metabolomic Analysis of Plasma Samples

Intending to highlight the metabolic similarity and differences between female and male basketball athletes, we determined their plasma metabolic profiles through GC–MS. A total of 50 different compounds were identified (Supplementary Table S1).

**Figure 3:**
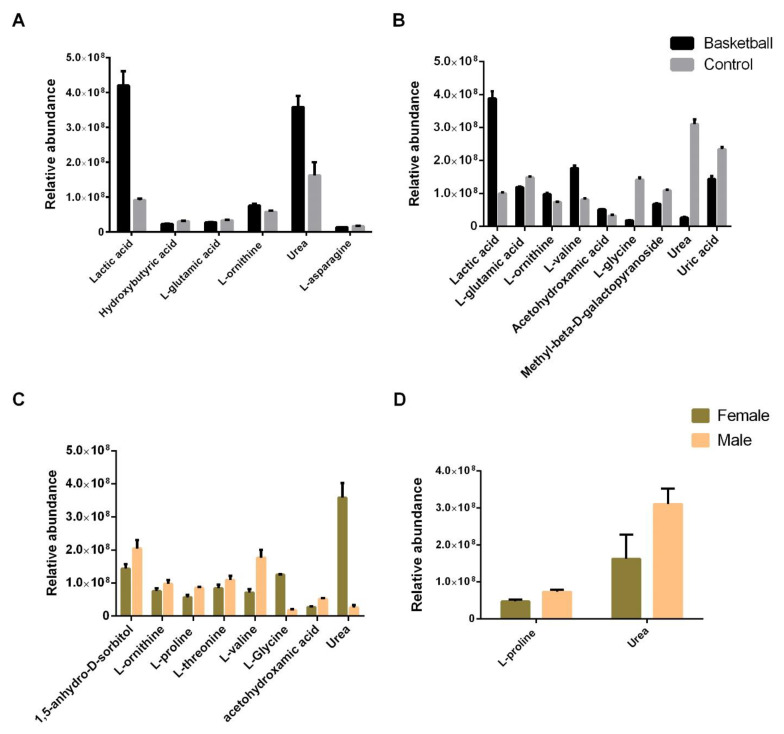
Plasma metabolomic profile of female and male basketball players using gas chromatography–mass spectrometry (GC–MS). (**A**) Histogram representation of plasma metabolites whose relative abundance is statistically different between female basketball athletes and controls (*p* < 0.05) (Militello et al., 2021). (**B**) Histogram representation of plasma metabolites whose relative abundance is statistically different between male basketball athletes and controls (*p* < 0.05). (**C**) Histogram representation of plasma metabolites whose relative abundance is statistically different between female and male basketball athletes (*p* < 0.05). (**D**) Histogram representation of plasma metabolites whose relative abundance is statistically different between female and male controls ( *p* < 0.05). Statistical analysis was performed by two-way ANOVA (Tukey’s multiple comparisons test) using GraphPad Prism 8.

In female athletes, compared to female controls, we found six statistically different concentrated metabolites (Figure 3A). Among them, lactic acid (460%, *p* = 0.0013; η^2^ = 0.937), urea (220%, *p* = 0.0361; η^2^ = 0.928), and ornithine (130%, *p* = 0.0467; η^2^ = 0.815) showed an increase in athletes compared to controls, while hydroxybutyric acid (75%, *p*= 0.0258; η^2^ = 0.641), L-glutamic acid (83%, *p* = 0.0353; η^2^ = 0.637), and L-asparagine (82%, *p* = 0.0498; η^2^ = 0.663) were statistically decreased.

In males, as reported in Figure 3B, we found nine metabolites differentially concentrated in the basketball players compared to the controls. Among them, lactic acid (389%, *p* = 0.0018; η^2^ = 0.937), acetohydroxamic acid (158%, *p* = 0.0081; η^2^ = 0.823), L-ornithine (132%, *p* = 0.0294; η^2^ = 0.815), and L- valine (218%, *p* = 0.0002; η^2^ = 0.936) were increased. On the contrary, L-glutamic acid (79%, *p* = 0.0467; η^2^ = 0.865), L-glycine (15%, *p* = 0.0001; η^2^ = 0.970), methyl-beta-D-galactopyranoside (63%, *p* = 0.0206; η^2^ = 0.660), urea (8%, *p* = 0.0005; η^2^ = 0.928), and uric acid (61%, *p* = 0.0091; η^2^ = 0.726) were decreased. Interestingly, L-isoleucine was found only in the male athletes.

Comparing the athletes, we found eight metabolites differentially concentrated in plasma. The amino acids L-ornithine (129%, *p* = 0.0472; η^2^ = 0.815), L-proline (149%, *p* = 0.0011; η^2^ = 0.911), L-threonine (130%, *p* = 0.0435; η^2^ = 0.643), and L-valine (247%, *p* = 0.0001; η^2^ = 0.936) were higher in males. Moreover, sorbitol (142%, *p* = 0.0128; η^2^ = 0.730) and acetohydroxamic acid (191%, *p* = 0.0017; η^2^ = 0.823) were more abundant in males. On the contrary, L-glycine (14%, *p* = 0.0001; η^2^ = 0.970) and urea (7%, *p* = 0.0004; η^2^ = 0.928) were lower in males compared to females.

With respect to the non-athlete subjects, we found that only L-proline (154%, *p* = 0.0021; η^2^ = 0.911) and urea (191%, *p* = 0.0201; η^2^ = 0.928) were differentially concentrated, and both were more abundant in males.

### 3.5. Steroid Hormones Evaluation in Plasma Samples

Nine steroid hormones were quantified in a LC–MS/MS single run by using an MRM mode on plasma samples. The hormonal levels of both male and female of athletes were compared to those of the relative control groups. The quantification of each steroid hormone was obtained by the interpolation of extracted ion chromatogram peak area onto the relative calibration curves built up for each analyte.

**Figure 4:**
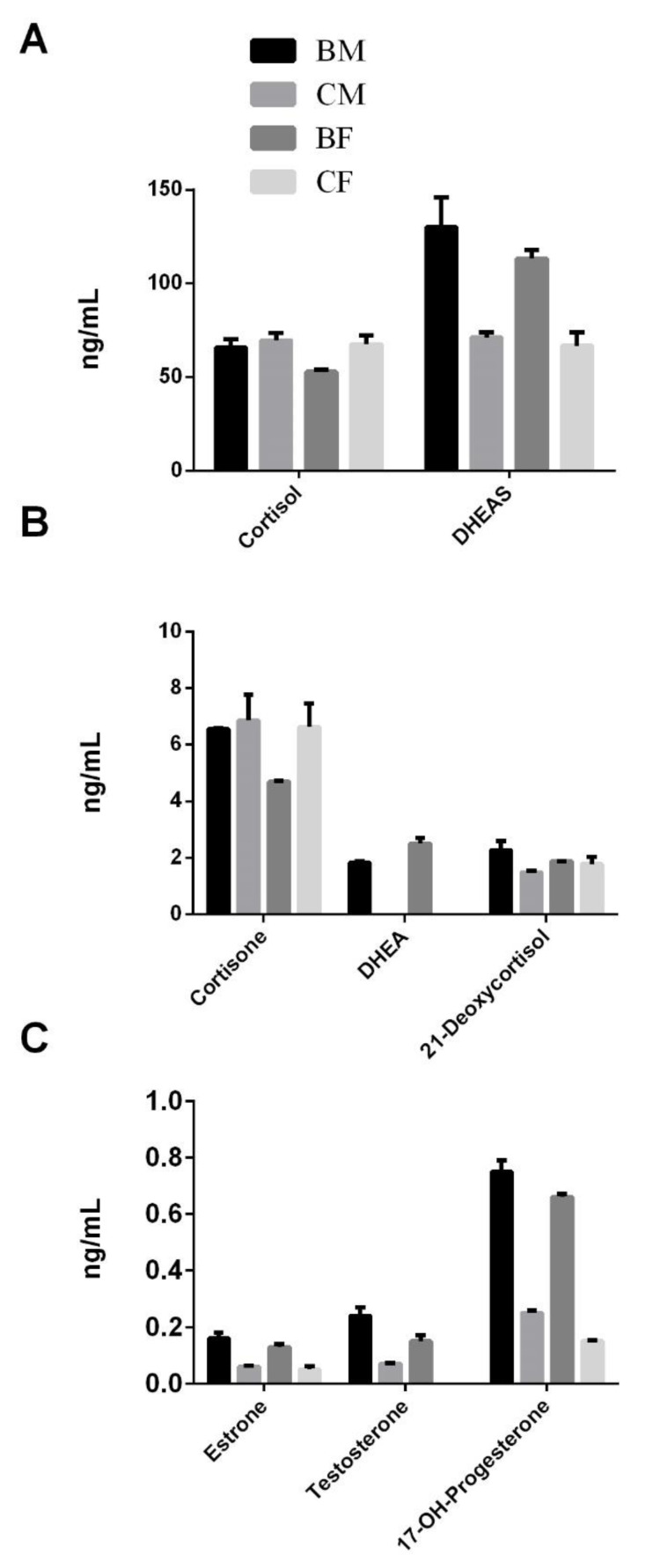
Measurements of steroid hormones quantified in the plasma of both male and female basketball players vs their respective controls. The histogram representations were separated in three panels (**A**–**C**) in agreement with the order of magnitude expressed in ng/mL. The statistical analysis was carried out by two-tailed *t*-test using GraphPad Prism 8, and only statistically significant differences were reported (*p* < 0.05).

Our samples indicated that dehydroepiandrosterone (DHEA) and its sulphate (DHEA-S; 182% for males, *p* = 0.0002; 169% for females, *p* = 0.0012; η^2^ = 0.9277) were enhanced in both male and female subjects who practice basketball, compared to the control groups (Figure 4A,B). Such increases enabled us to quantify the DHEA in the plasma of athletes after basketball activity, which was otherwise under the detection limit in the control subjects (Figure 4B). An analogue trend is observed for estrone (252% for males, *p* = 0.0001; 242% for females, *p* = 0.0002; η^2^ = 0.9549), testosterone (364% for males, *p* ≤ 0.05), and 17-OH-progesterone (303% for males, *p* = 0.0001; 451% for females, *p* = 0.0001; η^2^ = 0.9954), displaying a significant increase following physical activity (Figure 4C). Interestingly, the level of testosterone in the control females was below the limit of instrumental detection, whereas it was detected in females as a consequence of physical activity. Finally, cortisol, 21-deoxycortisol, cortisone, and estradiol showed a different trend in relation to sex: cortisol (78%, *p* = 0.0075; η^2^ = 0.8084), cortisone (70%, *p* = 0.0197; η^2^ = 0.7490), and estradiol (62%, *p* = 0.0016; η^2^ = 0.8298) decreased only in female basketball players, while there was no change in males, and 21-deoxycortisol (152%, *p* = 0.0094; η^2^ = 0.7197) increased in male athletes, but not in females (*p* ≤ 0.05; Figure 4A,B).

Comparing the athletes, cortisol (124%, *p* = 0.0161; η^2^ = 0.8084), cortisone (139%, *p* = 0.0252; η^2^ = 0.7490), 17-OH-progesterone (113%, *p* = 0.0038; η^2^ = 0.9954), and estradiol (133%, *p* = 0.0396; η^2^ = 0.8298) were higher in males.

Finally, the male controls had higher 17-OH-progesterone (166%, *p* = 0.0019; η^2^ = 0.9954) compared to the female controls.

### 3.6. Fatty Acids Evaluation in Plasma Samples

The quantification of plasma fatty acids was performed by the reaction of transesterification, converting triglycerides and free fatty acid in the corresponding FAMEs, followed by GC–MS analysis.

**Figure 5:**
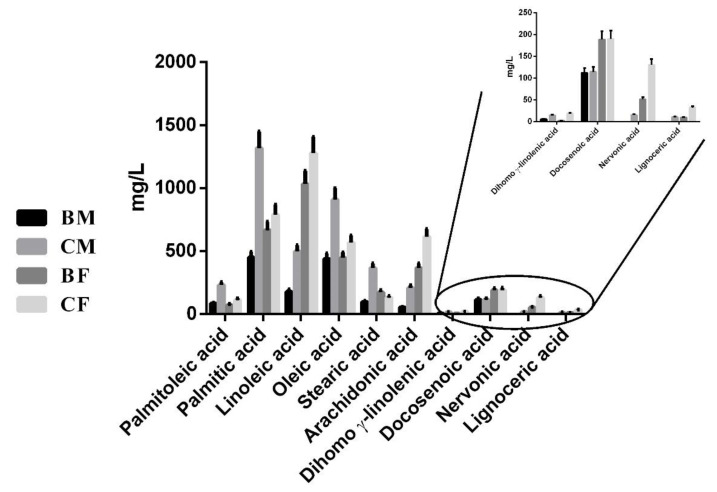
Comparison of the concentrations (expressed in mg/L) of FAME obtained by acid transesterification of the plasma from both male and female basketball players and from the control subjects. The statistical analysis was carried out by two-tailed *t*-test using GraphPad Prism 8 and only statistically significant differences were reported (*p* < 0.05).

Our results showed that there was a general trend of the identified fatty acids to decrease with physical activity, independent of sex. In particular, palmitoleic acid (36% for males, *p* = 0.0001; 65%, *p* = 0.0369 for females; η^2^ = 0.9680), palmitic acid (34% for males *p* = 0.001; 84% for females, *p* = 0.003; η^2^ = 0.9533), linoleic acid (35%, *p* = 0.0084 for males; 80%, *p* = 0.0332 for females; η^2^ = 0.9742), oleic acid (48% for males, *p* = 0.0001; 78%, *p* = 0.0157 for females; η^2^ = 0.9343), arachidonic acid (26%, *p* = 0.0047 for males; 59%, *p* = 0.0001 for females; η^2^ = 0.9788), dihomo γ-linolenic acid (41%, *p* = 0.0001 for males; 14%, *p* = 0.0001 for females; η^2^ = 0.9764), 15-tetracosenoic (nervonic) acid (39% for females), and tetracosanoic (lignoceric) acid (30% for females) decreased in both female and male basketball players compared to the control groups (*p* ≤ 0.05; Figure 5). Even two C24 fatty acids, e.g., 15-tetracosenoic (nervonic) acid and tetracosanoic (lignoceric) acid, decreased because of the physical activity to concentrations lower than the limit of instrumental detection in the male basketball players group. Interestingly, docosenoic acid was slightly more abundant in females than males, but it did not change in relation to sport (Figure 5), and stearic acid exhibited a different tendency in relation to physical activity and sex; in fact, it decreased in the male basketball players (26%, *p* = 0.0001; η^2^ = 0.9714) but increased in the female athletes (131%, *p* = 0.132; η^2^ = 0.9714) (Figure 5).

Comparing the athletes, linoleic acid (17%, *p* = 0.001; η^2^ = 0.9742), stearic acid (57%, *p* = 0.0123) η^2^ = 0.9714), arachidonic acid (15%, *p* = 0.0001; η^2^ = 0.9788), and docosenoic acid (59%, *p* = 0.0015; η^2^ = 0.8990) were lower in males.

There were also differences in the sedentary controls where, in fact, in male subjects, palmitoleic acid (206%, *p* = 0.0001; η^2^ = 0.9680), palmitic acid (166%, *p* = 0.003; η^2^ = 0.9533), oleic acid (160%, *p* = 0.0007; η^2^ = 0.9343), and stearic acid (278%, *p* = 0.0001; η^2^ = 0.9714) were higher than in female subjects. Instead, linoleic acid (30%, *p* = 0.0001; η^2^ = 0.9742), arachidonic acid (33%, *p* = 0.001; η^2^ = 0.9788), and docosenoic acid (60%; *p* = 0.0016; η^2^ = 0.8990) showed the opposite trend, decreasing in the male subjects.

### 3.7. Metabolic Pathways Analysis

To have an overview of the metabolic process involved in adaptation to physical activity, we used the plasma metabolites identified in this study, showing a statistically significant increase/decrease level in female and male basketball players in comparison with the relative sedentary controls. We used MetScape (http://metscape.med.umich.edu, accessed on 10 March 2021—from Cytoscape), which provides a bioinformatics tool for the interpretation of metabolomic data [26]. The results obtained are reported in Table 3. We identified 14 metabolic pathways in common between female and male athletes, including, among others, amino acids, fatty acids, and glycidic metabolism; 4 pathways typical of the female metabolism involving mainly fatty acids; and 6 pathways characteristic of males, including amino acids, fatty acids, and porphyrin metabolism (for details see Table 3).

## 4. Discussion

The purpose of this study was to compare the redox, hormonal, metabolic, and lipid profiles of female and male basketball players during the seasonal training period, compared to the relative sedentary control groups. Further, this investigation aimed to be descriptive and exploratory to deepen the understanding of biochemical alterations that occur in adaptation to physical exercise in female and male athletes, underlining their common and different traits. As the control, we planned to use untrained subjects because they represent the reference value of the analyzed parameters, trying to highlight the changes due only to regular physical activity.

In our experimental conditions, in female athletes with a body mass index comparable to controls, the antioxidant capacity showed a significant increase and a reduction in oxidative species. In males, we found a similar increase in the antioxidant capacity, although the oxidative species are stackable with the relative controls. Therefore, it is known that females had a greater oxidative status than males [27]. In our experimental conditions, we observed in both sexes a general increase in antioxidant capacity. As previously reported in our study, during physical activity there is an increase in ROS production due to the stress of physical exercise [2]. Consequently, the high levels of antioxidant capacity of female and male athletes attenuate this oxidant production. Several studies reported that in basketball players, oxidative stress at the end of the season is not severe, despite the high number of matches played, and this is probably thanks to the increases in the antioxidant defense mechanisms [28,29]. The same trends were observed for other sports, such as soccer, cycling, and swimming [2]. In this context, we suggest that in both sexes, adaptation to physical exercise leads to an increase in antioxidant capacity that is able to counteract the oxidative stress produced during exercise, guaranteeing health and better performance. However, high effort and excessive levels of oxidative stress can lead to a rise in inflammatory markers and overtraining syndrome [5].

Resistance exercise and/or training elicits a milieu of acute physiological responses and chronic adaptations that are critical for increasing muscular strength, power, hypertrophy, and local muscular endurance [30]. In our study, we analyzed several hormones, such as salivary cortisol, plasma adiponectin, and several steroid hormones. Salivary cortisol quantification revealed that both in females and in males, this hormone decreases with athletic activity. This agrees with other studies that reported a reduction of cortisol for both females and males after a match [31]. We also analyzed the same hormone in plasma, finding a comparable trend for females and males, although in males, this difference is not statistically significant. This divergence could be explained by the different techniques used and/or by the biological samples analyzed; in fact, other authors have reported that cortisol changes occurred simultaneously in plasma and saliva, but the timing of post-exercise hormone moving varied between trials and individuals [32]. Moreover, specific proteins binding cortisol affecting the circulating pool of bioactive free cortisol in plasma have been described [33].

In this study, we evaluated the levels of adiponectin by Western blot, highlighting its increase in male players. Similarly, Jurimae and colleagues found that in highly trained athletes, during the recovery period, adiponectin levels increased [34,35]. Contrarily, in females, we found that the plasma levels of this adipokine were not changed. Nevertheless, the adiponectin levels that we found in control females was higher than in control males, confirming the high levels of this adipokine in females [36,37]. Basketball is a sport more associated with fast twitch muscular fibres (type II) mainly involved in quick bursts with great power [16,38], than it is with slow twitch fibres (type I), which in turn are mainly involved in endurance sports. Interestingly, males expressed 42% more of this type of fibers than did females, which, in addition to showing a lower expression of AdipoR1 [37], suggests a sex-related difference in adiponectin susceptibility.

Finally, we evaluated the levels of several steroid hormones. We found an increase of testosterone, DHEA, estrone, and 17-OH-progesterone both in females and males. Testosterone increases muscle mass and strength over weeks to months, with a strong dose-response evident from below to above physiological testosterone doses and concentrations [16], and several reports have indicated its increase after exercise [39]. Likewise, other authors have recorded a significant increase of DHEA levels monitored in the sera of older athletes immediately after exercise [40]. In a similar manner, estrogens have been shown to reduce bone resorption and muscle damage, which may have important ramifications for musculoskeletal adaptations to resistance training [30]. On the other hand, estradiol decreases in athletes, regardless of sex, as reported by others, emphasizing the benefit of physical activity, especially for females to reduce the main risk factor for estrogen-responsive breast cancer [41].

To have a further overview of the process involved in female and male adaptation to physical exercise, we analyzed the plasma metabolic and lipid profiles of participants. Comparing players versus sedentary subjects, our results showed that there was a general trend of fatty acids decreasing with physical activity, independent of sex. This finding is in agreement with the activation of fatty acid metabolism during physical activity, where energy reservoirs of triacylglycerols stored within the adipose tissues were used as fuel for working muscles [42].

The MetScape program (version 3.1.3) allowed us to identify the main metabolic pathways implicated in physical exercise in females and males. These include amino acids, fatty acids, and glycidic metabolism, but also arachidonic acid metabolism as a response to the inflammation process [43]. Moreover, we identified the urea cycle, which is the main system for ammonia elimination and detoxification, for reducing fatigue and improving exercise endurance capacity [44]. Considering the pathways differentially involved among the sexes, in females we observed mainly lipid metabolism (such as butanoate, squalene, and others), while in males, we observed primarily amino acids metabolism, in particular the branched chain. Increases in serum BCAAs (branched chain amino acids) during or after exercise may indicate their mobilization from either liver or muscle [45]. In skeletal muscle, they promote glucose uptake and protein synthesis, thus playing an important role during exercise and especially in post-exercise recovery. Moreover, exercise increases the ability of mitochondria from skeletal muscle to oxidize BCAAs as an alternative source of energy without inducing insulin resistance [46].

Already, other researchers have highlighted significant sex-related differences in energy and macronutrient metabolism within many tissues and organs, resulting in a distinct metabolic profile between females and males and, in particular, the use of nutritional fats and the distribution of fat depots, glucose homeostasis, amino acid transport, and protein metabolism [47]. Moreover, sex differences in substrate metabolism during whole body moderate-to-high intensity endurance exercise have also been reported, with females shown to oxidize more fat and less carbohydrate than males when exercising at the same relative intensity [48,49,50]. Our obtained data suggest that adaptation to training increases this difference between male and female metabolisms.

Our analysis was performed 24 h after training sessions, and, interestingly, we observed a relevant increase in lactate in athletes. Lactate, once recognized as merely a product of glycogenolysis, is now seen as a carbohydrate fuel source [51,52] and a signaling molecule contributing to mitochondrial adaptations in skeletal muscle [53,54]. We think that it is a key regulator of adaptation, and, probably, the ability to produce and tolerate high levels of blood lactate during effort is associated with a successful performance.

Further investigations are compelling to elucidate the role of lactate in adaptation to physical exercise.

## 5. Limitation

The main limitation of this study is the lower number of participants belonging to the same teams. Furthermore, our data relates exclusively to basketball, as it is characterized by specific metabolic demands, and it cannot be transferred to all other sports. Therefore, this should be considered a pilot study. It will be interesting to evaluate if the same differences are also evident for sports with metabolic characteristics different from those of basketball as analyzed in this study.

Moreover, we did not consider the menstrual cycle phase of female participants. There are conflicting results on menstrual cycle phase effects on physical activity [55], with studies reporting improved performance outcomes during the early follicular [56], ovulatory [57], and mid-luteal [58] phases; whereas others have shown no changes in exercise performance between menstrual cycle phases [59,60,61].

## 6. Conclusions

Despite its limitations, our study indicates that female and male adaptations to training have several common traits, such as antioxidant potential enhancement, lactate elevation, and activation of detoxifying process such as the urea cycle and arachidonic pathways as a response to inflammation. We also highlighted some different sex-related behaviors, including dissimilar lipid and amino acids utilization, which need further investigation.

## Figures and Tables

**Table 1:** GC–MS parameters used for fatty acids analysis.

	Rate (°C/min)	Value (°C)	Hold Time (min)	Run Time (min)
(initial)		90	1	1
Ramp 1	10	140	2	8
Ramp 2	5	180	0	16
Ramp 3	10	280	5	31

**Table 2:** Participants’ characteristics.

Characteristics	Mean (SD)				Tukey’s Test ^a^			
	Basket Male	Control Male	Basket Female	Control Female	BM vs. CM	BF vs. CF	BM vs. BF	CM vs. CF
Age (year)	21 ± 2.2	26.1 ± 4.1	25.1 ± 5.5	26.9 ± 2.2	0.01 *	0.77	0.08	0.97
Weight (kg)	81.5 ± 10.2	73 ± 8.7	68.7 ± 11.9	58.7 ± 5.8	0.17	0.17	0.03 *	0.02 *
Height (cm)	186 ± 0.06	178.7 ± 0.06	175.6 ± 0.08	163.4 ± 0.06	0.06	0.003 **	0.006 **	<0.0001 ****
BMI (kg/m^2^)	23.6 ± 2.7	22.9 ± 2.9	22.1 ± 2.05	22 ± 2.3	0.64	1.00	0.18	0.72

^a^ Tukey’s test was performed by GraphPad Prism 8.0 software between the male basketball group (BM), male control group (CM), female basketball group (BF), and female control group (CF) (* *p* < 0.05; ** *p* < 0.01; and **** *p* < 0.0001).

**Table 3:** The selected metabolic pathways obtained using plasma metabolites identified in this study showing a statistically significant increase/decrease in the female and male basketball players in comparison with the relative sedentary controls. The pathways analysis was carried out using the MetScape 3 App for Cytoscape (http://metscape.med.umich.edu, accessed on 10 March 2021).

Metabolic Pathways Involved in Females and Males
Androgen and estrogen biosynthesis and metabolism
Arachidonic acid metabolism
Bile acid biosynthesis
C21-steroid hormone biosynthesis and metabolism
De novo fatty acid biosynthesis
Di-unsaturated fatty acid beta-oxidation
Glycolysis and gluconeogenesis
Histidine metabolism
Leukotriene metabolism
Linoleate metabolism
Omega-6 fatty acid metabolism
Purine metabolism
Urea cycle and metabolism of arginine, proline, glutamate, aspartate, and asparagine
Vitamin B9 (folate) metabolism
**Metabolic pathways involved only in females**
Butanoate metabolism
De novo fatty acid biosynthesis
Omega-6 fatty acid metabolism
Squalene and cholesterol biosynthesis
**Metabolic pathways involved only in males**
Glycine, serine, alanine, and threonine metabolism
Lysine metabolism
Porphyrin metabolism
Prostaglandin formation from arachidonate
Saturated fatty acids beta-oxidation
Valine, leucine, and isoleucine degradation

## Data Availability

The data presented in this study are available on request from the authors.

## Supplementary Materials

The following supporting information can be downloaded at: https://www.mdpi.com/article/10.3390/healthcare10020358/s1; Table S1: List of Metabolites identified in plasma samples of the subject utilized in this study by Gas Chromatography–mass spectrometry (GC-MS) analysis.

## References

[1] Hawley J.A., Lundby C., Cotter J.D., Burke L.M. (2018). Maximizing Cellular Adaptation to Endurance Exercise in Skeletal Muscle. Cell Metab..

[2] Luti S., Modesti A., Modesti P.A. (2020). Inflammation, Peripheral Signals and Redox Homeostasis in Athletes Who Practice Different Sports. Antioxidants.

[3] Hadžović-Džuvo A., Valjevac A., Lepara O., Pjanić S., Hadžimuratović A., Mekić A. (2014). Oxidative stress status in elite athletes engaged in different sport disciplines. Bosn. J. Basic Med. Sci..

[4] Mangine G.T., Hoffman J.R., Gonzalez A.M., Townsend J.R., Wells A.J., Jajtner A.R., Beyer K.S., Boone C.H., Wang R., Miramonti A.A. (2017). Exercise-Induced Hormone Elevations Are Related to Muscle Growth. J. Strength Cond. Res..

[5] Magherini F., Fiaschi T., Marzocchini R., Mannelli M., Gamberi T., Modesti P.A., Modesti A. (2019). Oxidative stress in exercise training: The involvement of inflammation and peripheral signals. Free Radic. Res..

[6] Martinez-Huenchullan S.F., Tam C.S., Ban L.A., Ehrenfeld-Slater P., Mclennan S.V., Twigg S.M. (2020). Skeletal muscle adiponectin induction in obesity and exercise. Metabolism.

[7] Ren Y., Li Y., Yan J., Ma M., Zhou D., Xue Z., Zhang Z., Liu H., Yang H., Jia L. (2017). Adiponectin modulates oxidative stress-induced mitophagy and protects C2C12 myoblasts against apoptosis. Sci. Rep..

[8] Cnop M., Havel P.J., Utzschneider K.M., Carr D.B., Sinha M.K., Boyko E.J., Retzlaff B.M., Knopp R.H., Brunzell J.D., Kahn S.E. (2003). Relationship of adiponectin to body fat distribution, insulin sensitivity and plasma lipoproteins: Evidence for independent roles of age and sex. Diabetologia.

[9] Böttner A., Kratzsch J., Müller G., Kapellen T.M., Blüher S., Keller E., Blüher M., Kiess W. (2004). Gender differences of adiponectin levels develop during the progression of puberty and are related to serum androgen levels. J. Clin. Endocrinol. Metab..

[10] Gamberi T., Magherini F., Fiaschi T. (2019). Adiponectin in Myopathies. Int. J. Mol. Sci..

[11] Agostinis-Sobrinho C., Santos R., Moreira C., Abreu S., Lopes L., Oliveira-Santos J., Rosário R., Póvoas S., Mota J. (2016). Association between serum adiponectin levels and muscular fitness in Portuguese adolescents: LabMed Physical Activity Study. Nutr. Metab. Cardiovasc. Dis..

[12] Senefeld J.W., Clayburn A.J., Baker S.E., Carter R.E., Johnson P.W., Joyner M.J. (2019). Sex differences in youth elite swimming. PLoS ONE.

[13] Handelsman D.J., Hirschberg A.L., Bermon S. (2018). Circulating Testosterone as the Hormonal Basis of Sex Differences in Athletic Performance. Endocr. Rev..

[14] Vesper H.W., Wang Y., Vidal M., Botelho J.C., Caudill S.P. (2015). Serum Total Testosterone Concentrations in the US Household Population from the NHANES 2011–2012 Study Population. Clin. Chem..

[15] Handelsman D.J., Sikaris K., Ly L.P. (2016). Estimating age-specific trends in circulating testosterone and sex hormone-binding globulin in males and females across the lifespan. Ann. Clin. Biochem..

[16] Handelsman D.J. (2017). Sex differences in athletic performance emerge coinciding with the onset of male puberty. Clin. Endocrinol..

[17] Hoffman J.R., Bar-Eli M., Tenenbaum G. (1999). An examination of mood changes and performance in a professional basketball team. J. Sports Med. Phys. Fit..

[18] McInnes S.E., Carlson J.S., Jones C.J., McKenna M.J. (1995). The physiological load imposed on basketball players during competition. J. Sports Sci..

[19] Ben Abdelkrim N., El Fazaa S., El Ati J. (2007). Time-motion analysis and physiological data of elite under-19-year-old basketball players during competition. Br. J. Sports Med..

[20] Scanlan A., Dascombe B., Reaburn P. (2011). A comparison of the activity demands of elite and sub-elite Australian men’s basketball competition. J. Sports Sci..

[21] Hagströmer M., Oja P., Sjöström M. (2006). The International Physical Activity Questionnaire (IPAQ): A study of concurrent and construct validity. Public Health Nutr..

[22] Foster C., Florhaug J.A., Franklin J., Gottschall L., Hrovatin L.A., Parker S., Doleshal P., Dodge C. (2001). A new approach to monitoring exercise training. J. Strength Cond. Res..

[23] Militello R., Luti S., Parri M., Marzocchini R., Soldaini R., Modesti A., Modesti P.A. (2021). Redox Homeostasis and Metabolic Profile in Young Female Basketball Players during in-Season Training. Healthcare.

[24] Luti S., Fiaschi T., Magherini F., Modesti P.A., Piomboni P., Governini L., Luddi A., Amoresano A., Illiano A., Pinto G. (2020). Relationship between the metabolic and lipid profile in follicular fluid of women undergoing in vitro fertilization. Mol. Reprod. Dev..

[25] McGuigan M.R., Egan A.D., Foster C. (2004). Salivary Cortisol Responses and Perceived Exertion during High Intensity and Low Intensity Bouts of Resistance Exercise. J. Sports Sci. Med..

[26] Gao J., Tarcea V.G., Karnovsky A., Mirel B.R., Weymouth T.E., Beecher C.W., Cavalcoli J.D., Athey B.D., Omenn G.S., Burant C.F. (2010). Metscape: A Cytoscape plug-in for visualizing and interpreting metabolomic data in the context of human metabolic networks. Bioinformatics.

[27] Wiecek M., Maciejczyk M., Szymura J., Szygula Z. (2015). Changes in Oxidative Stress and Acid-Base Balance in Men and Women Following Maximal-Intensity Physical Exercise. Physiol. Res..

[28] Spanidis Y., Goutzourelas N., Stagos D., Mpesios A., Priftis A., Bar-Or D., Spandidos D.A., Tsatsakis A.M., Leon G., Kouretas D. (2016). Variations in oxidative stress markers in elite basketball players at the beginning and end of a season. Exp. Ther. Med..

[29] Yilmaz N., Erel Ö., Hazer M., Bagci C., Namiduru E., Giil E. (2007). Biochemical assessments of retinol, α-tocopherol, pyridoxal—5-phosphate oxidative stress index and total antioxidant status in adolescent professional basketball players and sedentary controls. Int. J. Adolesc. Med. Health.

[30] Kraemer W.J., Ratamess N.A. (2005). Hormonal responses and adaptations to resistance exercise and training. Sports Med..

[31] Casanova N., Palmeira-DE-Oliveira A., Pereira A., Crisóstomo L., Travassos B., Costa A.M. (2016). Cortisol, testosterone and mood state variation during an official female football competition. J. Sports Med. Phys. Fit..

[32] Tanner A.V., Nielsen B.V., Allgrove J. (2014). Salivary and plasma cortisol and testosterone responses to interval and tempo runs and a bodyweight-only circuit session in endurance-trained men. J. Sports Sci..

[33] Schwinn A.-C., Sauer F.J., Gerber V., Bruckmaier R.M., Gross J.J. (2018). Free and bound cortisol in plasma and saliva during ACTH challenge in dairy cows and horses. J. Anim. Sci..

[34] Jurimae J., Purge P., Jurimae T. (2005). Adiponectin is altered after maximal exercise in highly trained male rowers. Eur. J. Appl. Physiol..

[35] Plinta R., Olszanecka-Glinianowicz M., Drosdzol-Cop A., Chudek J., Skrzypulec-Plinta V. (2012). The effect of three-month pre-season preparatory period and short-term exercise on plasma leptin, adiponectin, visfatin, and ghrelin levels in young female handball and basketball players. J. Endocrinol. Investig..

[36] Waki H., Yamauchi T., Kamon J., Ito Y., Uchida S., Kita S., Hara K., Hada Y., Vasseur F., Froguel P. (2003). Impaired multimerization of human adiponectin mutants associated with diabetes. Molecular structure and multimer formation of adiponectin. J. Biol. Chem..

[37] Høeg L.D., Sjøberg K.A., Lundsgaard A.-M., Jordy A.B., Hiscock N., Wojtaszewski J.F.P., Richter E.A., Kiens B. (2013). Adiponectin concentration is associated with muscle insulin sensitivity, AMPK phosphorylation, and ceramide content in skeletal muscles of men but not women. J. Appl. Physiol..

[38] Senefeld J., Joyner M.J., Stevens A., Hunter S.K. (2016). Sex differences in elite swimming with advanced age are less than marathon running. Scand. J. Med. Sci. Sports.

[39] Vingren J.L., Kraemer W.J., Ratamess N.A., Anderson J.M., Volek J.S., Maresh C.M. (2010). Testosterone physiology in resistance exercise and training: The up-stream regulatory elements. Sports Med..

[40] Heaney J.L.J., Carroll D., Phillips A.C. (2013). DHEA, DHEA-S and cortisol responses to acute exercise in older adults in relation to exercise training status and sex. Age.

[41] Ennour-Idrissi K., Maunsell E., Diorio C. (2015). Effect of physical activity on sex hormones in women: A systematic review and meta-analysis of randomized controlled trials. Breast Cancer Res. BCR.

[42] Mika A., Macaluso F., Barone R., Di Felice V., Sledzinski T. (2019). Effect of Exercise on Fatty Acid Metabolism and Adipokine Secretion in Adipose Tissue. Front. Physiol..

[43] Wang T., Fu X., Chen Q., Patra J.K., Wang D., Wang Z., Gai Z. (2019). Arachidonic Acid Metabolism and Kidney Inflammation. Int. J. Mol. Sci..

[44] Chen S., Minegishi Y., Hasumura T., Shimotoyodome A., Ota N. (2020). Involvement of ammonia metabolism in the improvement of endurance performance by tea catechins in mice. Sci. Rep..

[45] Shimomura Y., Murakami T., Nakai N., Nagasaki M., Harris R.A. (2004). Exercise promotes BCAA catabolism: Effects of BCAA supplementation on skeletal muscle during exercise. J. Nutr..

[46] Shou J., Chen P.-J., Xiao W.-H. (2019). The Effects of BCAAs on Insulin Resistance in Athletes. J. Nutr. Sci. Vitaminol..

[47] Comitato R., Saba A., Turrini A., Arganini C., Virgili F. (2015). Sex Hormones and Macronutrient Metabolism. Crit. Rev. Food Sci. Nutr..

[48] Carter S.L., Rennie C., Tarnopolsky M.A. (2001). Substrate utilization during endurance exercise in men and women after endurance training. Am. J. Physiol. Endocrinol. Metab..

[49] Roepstorff C., Steffensen C.H., Madsen M., Stallknecht B., Kanstrup I.-L., Richter E.A., Kiens B. (2002). Gender differences in substrate utilization during submaximal exercise in endurance-trained subjects. Am. J. Physiol. Endocrinol. Metab..

[50] Roepstorff C., Thiele M., Hillig T., Pilegaard H., Richter E.A., Wojtaszewski J.F.P., Kiens B. (2006). Higher skeletal muscle alpha2AMPK activation and lower energy charge and fat oxidation in men than in women during submaximal exercise. J. Physiol..

[51] Brooks G.A. (2018). The Science and Translation of Lactate Shuttle Theory. Cell Metab..

[52] Ferguson B.S., Rogatzki M.J., Goodwin M.L., Kane D.A., Rightmire Z., Gladden L.B. (2018). Lactate metabolism: Historical context, prior misinterpretations, and current understanding. Eur. J. Appl. Physiol..

[53] Kitaoka Y., Takeda K., Tamura Y., Hatta H. (2016). Lactate administration increases mRNA expression of PGC-1α and UCP3 in mouse skeletal muscle. Appl. Physiol. Nutr. Metab..

[54] Hashimoto T., Hussien R., Oommen S., Gohil K., Brooks G.A. (2007). Lactate sensitive transcription factor network in L6 cells: Activation of MCT1 and mitochondrial biogenesis. FASEB J..

[55] McNulty K.L., Elliott-Sale K.J., Dolan E., Swinton P.A., Ansdell P., Goodall S., Thomas K., Hicks K.M. (2020). The Effects of Menstrual Cycle Phase on Exercise Performance in Eumenorrheic Women: A Systematic Review and Meta-Analysis. Sports Med..

[56] Pallavi L.C., Souza U.J.D., Shivaprakash G. (2017). Assessment of Musculoskeletal Strength and Levels of Fatigue during Different Phases of Menstrual Cycle in Young Adults. J. Clin. Diagn. Res. JCDR.

[57] Bambaeichi E., Reilly T., Cable N.T., Giacomoni M. (2004). The isolated and combined effects of menstrual cycle phase and time-of-day on muscle strength of eumenorrheic females. Chronobiol. Int..

[58] Oosthuyse T., Bosch A.N., Jackson S. (2005). Cycling time trial performance during different phases of the menstrual cycle. Eur. J. Appl. Physiol..

[59] McLay R.T., Thomson C.D., Williams S.M., Rehrer N.J. (2007). Carbohydrate loading and female endurance athletes: Effect of menstrual-cycle phase. Int. J. Sport Nutr. Exerc. Metab..

[60] Janse DE Jonge X.A.K., Thompson M.W., Chuter V.H., Silk L.N., Thom J.M. (2012). Exercise performance over the menstrual cycle in temperate and hot, humid conditions. Med. Sci. Sports Exerc..

[61] Vaiksaar S., Jürimäe J., Mäestu J., Purge P., Kalytka S., Shakhlina L., Jürimäe T. (2011). No effect of menstrual cycle phase and oral contraceptive use on endurance performance in rowers. J. Strength Cond. Res..

