# Supplementary material for: Relationships between Sex and Adaptation to Physical Exercise in Young Athletes: A Pilot Study
Source: Healthcare (Basel). 2022 Feb 11;10(2):358. doi: 10.3390/healthcare10020358 (PMC8871996; doi:10.3390/healthcare10020358)
Supplement: Supplementary file 1 [file healthcare-10-00358-s001.zip › healthcare-1500232-supplementary.pdf]

Supplementary Table 1

Table S1. List of Metabolites identified in plasma samples of the subject utilized in this study by Gas Chromatography–mass spectrometry (GC-MS) analysis.

Metabolite number	Metabolite name	Retention Time	CAS number*	KEGG ID°
1	2-hydroxybutyric acid	[7.852]	565-70-8	C05984
2	L-lactic acid	[6.851]	79-33-4	C00186
3	L-asparagine	[14.984]	70-47-3	C00152
4	L-glutamic acid	[14.398]	56-86-0	C00025
5	L-ornithine	[16.632]	70-26-8	C00077
6	Acetohydroxamic acid	[7.72]	546-88-3	C06808
7	Urea	[9.599]	57-13-6	C00086
8	1,3-propanediol	[6.777]	504-63-2	C02457
9	Elaidic acid	[20.508]	112-79-8	C01712
10	Thymol	[10.475]	89-83-8	
11	1,5-anhydro-D-sorbitol	[16.967]	154-58-5	C07326
12	1,6-anhydro-glucose	[15.326]	498-07-7	C06478
13	1-octadecene	[16.36]	112-88-9	
14	2-amino-2-methyl-1,3-propanediol	[10.56]	115-69-5	C11260
15	D-galactose	[17.662]	59-23-4	C00124
16	D-mannitol	[17.81]	87-78-5	C00392
17	L-alanine	[7.4748]	56-41-7	C00041
18	L-glutamic acid (dehydrated)	[13.232]	56-86-0	C00025
19	L-glutamine	[13,431]	56-85-9	C00064
20	L-glutamine	[14,083]	56-85-9	C00064
21	L-glutamine	[16.092]	56-85-9	C00064
22	L-proline	[10.321]	147-85-3	C00148
23	L-serine	[11.174]	56-45-1	C00065
24	L-threonine 2	[11.464]	72-19-5	C00188
25	L-valine	[9.151]	72-18-4	C00183
26	L-aspartic acid	[13.207]	56-84-8	C00049
27	Citric acid	[16.615]	5949-29-1	C00158
28	Diglycerol	[15.958]	627-82-7	
29	Glyceric acid	[10.735]	473-81-4	C00258
30	L-glycine	[10.456]	56-40-6	C00037
31	Isopropyl beta-D-1-thiogalactopyranoside	[19.097]	367-93-1	C03619 C02327
32	Methyl-beta-D-galactopyranoside	[16.935]	1824-94-8	C03619
33	Oxalic acid	[7.883]	144-62-7	C00209
34	Porphine	[10.77]	681295-24-9	C09541
35	Ribose	[15.113]	50-69-1	C00121
36	Threonic acid	[13.652]	7306-96-9	C01620
37	Uric acid	[19.331]	66-22-8	C00366
38	Xanthotoxin	[20.715]	298-81-7	C01864
39	DL-isoleucine	[10.225]	443-79-8	C16434
40	Tyrosine	[17.871]	60-18-4	C00082
41	D-allose 1	[17.278]	579-36-2	C01487
42	L- sorbose	[17.187]	3615-56-3	C00247
43	L-lysine	[17.643]	56-87-1	C00047

44	L-threonine	[11.464]	72-19-5	C00188
45	citraconic acid	[10.792]	498-23-7	C02226
46	myo-inositol	[19.354]	87-89-8	C00137
47	eicosapentaenoic acid	[24.013]	10417-94-4	C06428
48	glycerol	[9.941]	56-81-5	C00116
49	ethanolamine	[9.879]	141-43-5	C00189
50	phosphoric acid	[9.966]	7664-38-2	C00009

* Chemical Abstract Service number. KEGG identifier (https://www.genome.jp/kegg/).
